# Clinical validity of the 12-item WHODAS-2.0 in a naturalistic sample of outpatients with psychotic disorders

**DOI:** 10.1186/s12888-021-03101-9

**Published:** 2021-03-10

**Authors:** Christopher Holmberg, Andreas Gremyr, Jarl Torgerson, Kirsten Mehlig

**Affiliations:** 1 Institute of Health and Care Sciences, University of Gothenburg, Arvid Wallgrens Backe, Box 457, 405 30 Göteborg, Sweden; 2 Department of Psychotic Disorders, Sahlgrenska University Hospital, Gothenburg, Sweden; 3 Jönköping Academy for Improvement of Health and Welfare, Jönköping University, Jönköping, Sweden; 4 School of Public Health and Community Medicine, Institute of Medicine, University of Gothenburg, Gothenburg, Sweden

**Keywords:** Assessment, Disability, IFC, PANSS, Psychometrics, Psychotic disorders, Validity, WHODAS-2.0

## Abstract

**Background:**

The World Health Organization Disability Assessment Schedule 2.0 (WHODAS-2.0) is a self-administered instrument to assess functional impairment. It is used in the general population as well as different patient groups. However, its application to patients with psychotic disorders may be hampered by disease-specific difficulties of self-estimation. This study aimed to examine the psychometric properties of the short (12-item) WHODAS-2.0 in a naturalistic sample of outpatients attending a psychosis clinic in Gothenburg, Sweden.

**Methods:**

Annual data from two outpatient clinics registered 2016–2019 were analyzed retrospectively. The assessment of the short WHODAS-2.0 was based on the first questionnaire completed by 881 patients. Confirmatory factor analysis evaluated previously validated models. Item convergent and discriminant validity as well as internal reliability were computed. Construct validity was assessed by comparing mean differences in accord with previous research regarding patients’ characteristics associated with functioning such as advanced age, diagnosed comorbidities, antipsychotic treatment status, and symptom severity measured with PANSS-8 remission items.

**Results:**

A heterogeneous sample was obtained in terms of age (range: 20–92), various living situations, and different geographic areas of birth. Most patients (75%) had been diagnosed with psychotic disorders more than 10 years ago and the majority (89%) were on antipsychotic medication. We confirmed an adjusted two-level factor model with a single second-order disability factor and six first-order factors representing the six IFC dimensions. The WHODAS-2.0 sum score measuring general disability showed good reliability (Cronbach's alpha = 0.89). Construct validity was confirmed as older patients, patients with comorbidities, and patients in assisted living had higher WHODAS-2.0 scores. Patients with no or mild psychotic symptoms had significantly lower WHODAS-2.0 sum scores than patients with more severe symptoms.

**Conclusions:**

The findings further validate the 12-item WHODAS-2.0 in a naturalistic sample of outpatients with psychotic disorders. This study corroborates the clinical significance of the short, 12-item WHODAS-2.0 by demonstrating consistent associations between patients’ age, medical comorbidities, living situation, antipsychotic treatment status, and psychotic symptom severity.

**Supplementary Information:**

The online version contains supplementary material available at 10.1186/s12888-021-03101-9.

## Background

The World Health Organization Disability Assessment Schedule 2.0 (WHODAS-2.0) is a tool to measure disability and functional impairment [1]. The WHODAS-2.0 is grounded in the conceptual framework of the International Classification of Functioning, Disability, and Health (ICF) and captures an individual’s level of functioning in six main domains: (a) understanding and communicating; (b) getting around (capacity to move one’s body); (c) self-care (ability to attend to personal hygiene, dressing and eating, and to live independently); (d) getting along (ability to interact with others); (e) life activities (ability to carry out responsibilities at home, work, and school); (f) and participation in society (capacity to engage in community, civil, and recreational activities) [1].

There has been an increased use of WHODAS since 2013 when the DSM-5 was introduced, which recommends the use of WHODAS-2.0 for the assessment of disability in adults with psychiatric disorders [2]. In this study, we focus on a sample of outpatients with psychotic disorders (e.g., schizophrenia, schizoaffective disorder). Individuals with psychotic disorders are more vulnerable than the general population to physical illness and disability due to psychosocial difficulties and an increased prevalence of obesity and comorbidities [3–5]. Cognitive, functional, and symptomatic disabilities might restrict lifestyle-related activities (e.g., diet, physical activity, health care) with health risk, which is amplified by low rates of medical screening, monitoring, and intervention [6]. Considering these increased risks of disability, validated instruments that accurately identify the level of disability are essential to implementing suitable interventions [5].

In this context, WHODAS-2.0, which is self-administered, appears suitable as it captures the subjective experience of the patients and might narrow potential gaps between clinicians’ and patients’ perspectives [5]. There has been an increasing focus in healthcare on patient-reported outcome measures (PROM) and how they can support better care [7, 8]. However, self-assessment of symptom measures do not have a strong tradition in schizophrenia research because of objections to their clinical validity, which contrasts with their popularity in other mental disorders such as depression and anxiety [5, 9]. For example, WHODAS-2.0 might lack clinical validity if the patient is experiencing psychotic symptoms at the time of administration. A study found that modifications to WHODAS-2.0 scores were necessary in most participants because of their clinical presentations [10]. Thus, it is important to examine how clinically assessed symptom severity relates to self-assessed WHODAS-2.0 scores.

The most widely used form of the WHODAS-2.0 is the 36-item structured interview version, which takes approximately 20 min to complete and has excellent psychometric properties [1, 2]. However, the 36-item version is not always feasible in typical clinical situations and there has been a need for shorter versions. This study examines the psychometric properties of the shorter, 12-item self-assessment questionnaire version of the WHODAS-2.0.

### Clinical validation of the WHODAS-2.0, 12-item version

In the WHODAS-2.0 field trials, the reduced 12-item scale, despite only taking around five minutes to complete, accounted for roughly 80% of the variance in the 36-item scale [1, 2]. Several large-scale studies have suggested that the 12-item WHODAS-2.0 is generally reliable and valid when administered as an interview [11, 12] or self-assessment questionnaire [12, 13]. However, studies have shown mixed results regarding the factor structure of the WHODAS-2.0 12-item version, and both a one-factor structure and a six-factor model specifying the six WHODAS-2.0 domains of functioning have been suggested [2]. A previous Swedish study using patients with affective disorders has not been able to verify the common factor structures, reporting high internal consistency but weak construct validity [14].

The official Swedish translation of WHODAS-2.0 was published by the Swedish National Board of Health and Welfare in 2015 [15]. The decision to use and translate the instrument was based on validation studies conducted in other countries in collaboration with the WHO. However, the Swedish version of the 12-item WHODAS-2.0 has not yet been validated in patients with psychotic disorders.

Several issues have been raised related to other psychometric properties. Many psychiatric studies use samples with rigid inclusion and exclusion criteria to gather homogenous data. While this reduced variation may yield high internal consistency, it is not usually obtained in naturalistic samples. Thus, this level of control comes at the potential cost of creating artificial situations, which might lack clinical relevance [16].

Several WHODAS-2.0 validation studies have evaluated construct validity by using self-reported measures such as quality of life questionnaires [2, 10]. Few studies have evaluated construct validity by means of objective measures such as clinical diagnosis of disease that would be expected to correlate with disability [2, 17]. This method is valuable since self-assessed instruments are prone to subjectivity and reporting bias, and to only correlate self-assessed measures may not be a reliable basis for evaluating construct validity.

This study therefore aimed to examine the psychometric properties of the 12-item WHODAS-2.0 in a naturalistic sample of outpatients in a Swedish psychosis clinic to inform the assessment tool’s clinical use.

## Methods

### Design

This was a psychometric evaluation of the 12-item WHODAS-2.0, which is administered annually to the hospital's outpatients with psychotic disorders per clinical routine. The design was a cross-sectional observational register-based study.

### Setting

The Department of Psychotic Disorders at Sahlgrenska University Hospital in Gothenburg, Sweden delivers specialized care for individuals with psychotic disorders in the area (population of around 600,000 people). It serves roughly 2600 patients, with schizophrenia spectrum disorder being the most common diagnosis at its seven outpatient units. About 20% of these patients need care at one of the department’s inpatient wards each year.

### Procedure

From patient records, background information was collected as well as information from the annual check-ups, which included the WHODAS-2.0 12-item questionnaire. The annual check-ups are conducted at outpatient clinics and focus on the patient’s mental and physical health.

The patient’s case manager is responsible for the annual check-up and interviews the patient regarding their health status and lifestyle (e.g., dietary habits, smoking status), administers the WHODAS-2.0 12-item questionnaire, and assesses the patient’s symptoms by means of the eight diagnostic-specific core symptoms from the Positive and Negative Syndrome Scale (PANSS) [16]. A physician conducts a medical examination by evaluating anthropometric and vital measures such as body weight, blood pressure, and ECG.

### Data collection

For this study, two outpatient clinics were chosen for data collection because they had piloted a ‘digital dashboard’ that enabled easier data collection with digital questionnaires and visualization of results to help patients and healthcare professionals to jointly assess progress. The details of this ‘digital dashboard’ project are described elsewhere [18].

There are about 400–500 patients in total that get care at the two outpatient clinics. We included all patients with WHODAS-2.0 values registered between January 2016 and December 2019. The annual check-up in its current form was first implemented in 2016. The number of patients with a complete annual check-up including background information and WHODAS-2.0 varied between 40 and 90% during those years according to clinic statistics.

A total of 1347 WHODAS-2.0 questionnaires were registered between 2016 and 2019. Of these, 225 had missing values for at least one of the items and were excluded from the analysis. The number of missing values ranged from 0 for item S1 to 65 for item S12. Of the remaining 1122 observations, the earliest registered values from each patient were kept, resulting in 881 unique observations.

Supplementary file 1 presents a flowchart of the sampling process and a comparison between observations with complete and incomplete WHODAS-2.0 values.

### Measures

Patient background information included: age (in years); sex; year of psychosis diagnosis; living situation (independently in regular housing conditions, regular housing conditions with home care assistance, special housing with care staff assistance, social housing, or homeless) educational attainment (six options ranging from ‘not completed junior high school’ to ‘completed university degree’); geographic area of birth (Sweden, Nordic countries, rest of Europe, and other continents); medical comorbidities (diagnosed with COPD, diabetes mellitus, CVD, thyroid disease, or kidney disease); and currently treated with antipsychotic medication (yes/no).

The self-administered 12-item WHODAS-2.0 includes 12 items covering different domains of functioning (Table 1). Patients were asked to consider the past 30 days when answering the questions [19]. Each item used a five-level scale with 1 denoting “no difficulty” and 5 denoting “extreme difficulty or cannot do.” The simple sum of item scores across all domains constitutes a statistic that is sufficient to describe the degree of functional limitations [1, 19], whereas the subscales provide information on specific domains of functional impairment [1].

**Table 1:** WHODAS-2.0, 12-item version: to what extent are you able to fulfill the following tasks?

Item	A priori dimension^**a**^
S1 Standing for long periods such as 30 min?	Mobility (getting around)
S7 Walking a long distance such as a kilometer or equivalent?	Mobility (getting around)
S8 Washing your whole body?	Self-care
S9 Getting dressed?	Self-care
S2 Taking care of your household responsibilities?	Household (life activities)
S12 Your day-to-day work/school?	Household (life activities)
S3 Learning a new task, such as learning how to get to a new place?	Cognitive (understanding and communicating)
S6 Concentrating on doing something for ten minutes?	Cognitive (understanding and communicating)
S10 Dealing with people you do not know?	Social (getting along with people)
S11 Maintaining a friendship?	Social (getting along with people)
S4 How much of a problem do you have joining in community activities (for example, festivities) in the same way as anyone else can?	Society (participation in society)
S5 How much have you been emotionally affected by your health problems?	Society (participation in society)

^a^ As outlined in the 36-item version

The eight diagnostic-specific core symptoms from the Positive and Negative Syndrome Scale (PANSS) [20], assessed by case managers at the annual check-ups, were used to investigate whether patients with no or mild symptoms would show different WHODAS scores compared with patients having moderate to extreme symptoms. The items represent delusions (item P1), unusual thought content (G9), hallucinatory behavior (P3), conceptual disorganization (P2), mannerisms and posturing (G5), blunted affect (N1), social withdrawal (N4), and lack of spontaneity (N6) [20, 21]. The scores include 1- absent, 2- minimal, 3- mild, 4- moderate, 5- moderate-severe 6- severe, 7- extreme. Clinical remission is defined as 3 or less (mild symptom intensity) for at least six months, for all eight core items [20]. The PANSS-8-items have been validated in both clinical trials and clinical practice [22, 23].

### Statistical methods

Factor analytic approaches were used to explore the validity of the WHODAS-2.0 in the study population. We chose confirmatory factor analysis (CFA) because we had a theoretical framework and previously proposed models [11, 12, 24]. We assessed the overall fit of one strong disability factor (all 12 items loading on a single factor). This one-factor model was compared with a model including six first-order factors representing each two-item subscale of the WHODAS-2.0, which has been supported by previous research [11, 12].

Maximum likelihood estimation was used since the WHODAS-2.0 sum score was normally distributed (skewness < 2.00, Kurtosis < 7.00) and because we had a large sample size [25]. Based on recommendations [24], the a priori criteria for an acceptable model were the comparative fit index (CFI) > 0.90, Tucker-Lewis index (TLI) > 0.90, root mean square error of approximation (RMSEA) ≤ 0.08, and standardized root mean square residual (SRMR) <  0.08. Because the chi-squared test statistic is strongly affected by sample size, the normed chi-square (NC) was calculated by dividing the chi-squared value by the model’s degrees of freedom, with NC < 5.0 considered acceptable [26]. Model fit was further improved by including covariance parameters between item error terms based on modification indices provided in AMOS/SAS CALIS.

Item convergent and discriminant validity were assessed by calculating Spearman’s correlation coefficient for items within and between the six domains. Convergent validity indicates whether measures of the same construct are correlated, and discriminant validity assesses whether two measures are unrelated. A correlation coefficient of .75 or more indicates a strong relationship, .50–.74 indicates a moderate relationship, and .49 or less indicates a weak relationship [27].

Internal reliability was assessed using Cronbach’s alpha and supplemented with Spearman-Brown scores, as these are better for assessing internal reliability between scales with only two items [28]. Cronbach’s alpha ≥0.9 is usually regarded as excellent, 0.8–0.9 as good, 0.6–0.7 as acceptable, and ≥ 0.6 as questionable [29].

Construct validity should be assessed by testing predefined hypotheses about expected correlations with objective measures for the proposed construct [30]. In the context of psychotic disorders, we know that age, comorbidities such as cardiovascular disease (CVD) and chronic obstructive pulmonary disease (COPD), assisted living, and antipsychotic medication are associated with the level of disability [5, 6, 17]. Similarly, from previous research, we know that the severity of psychotic symptoms correlates with the level of psychosocial disability [5].

We hypothesized that high age (using the median 53 years as the cut-off), the presence of at least one clinically diagnosed medical comorbidity (COPD, diabetes mellitus, CVD, thyroid disease, or kidney disease), and assisted living status would correlate to a higher degree of disability, indicated by higher mean total WHODAS-2.0 scores. We also hypothesized that current use of antipsychotic medications would correlate to higher WHODAS-2.0 scores as these medications are associated with side effects such as rigidity and metabolic complications, and that patients not currently treated with medication might be in symptom remission, which in turn would correlate to lower levels of disability. Furthermore, we hypothesized that patients with high PANSS-8-item scores (> median 14 on the sum score, or > 3 per individual item), indicating moderate to extreme psychotic symptoms, would have higher WHODAS-2.0 scores compared with patients with lower PANSS-8-item scores. To test these hypotheses, we performed subgroup analyses using independent samples t-tests and the Mann-Whitney U test.

SPSS v.26 (IBM), AMOS v.26 (IBM), and SAS v.9.4 CALIS Procedure were used for all statistical analyses.

### Ethical considerations

The study used already registered patient data. Ethical approval was granted by the Swedish Ethical Review Authority (#2020–03010).

## Results

### Sample characteristics

The sample contained a similar distribution of male and female patients. Most of them, 657 (75%), were diagnosed with psychotic disorders more than 10 years ago (Table 2).

**Table 2:** Sample characteristics

Characteristics	Sample (***n*** = 881)
Age (years), mean (SD), range	52 (13.8), 20–92
Female, n (%)	413 (47%)
Male, n (%)	468 (53%)
Currently on antipsychotic medication, n (%)	
Yes	782 (89%)
No	61 (7%)
Missing information	38 (4%)
Living situation, n (%)	
Independently in regular housing	477 (54%)
Regular housing with care assistance	212 (24%)
Permanent special housing with care staff	143 (16%)
Other living arrangements or homeless	25 (3%)
Missing information	24 (3%)
Geographic birth area, n (%)	
Sweden	604 (69%)
Other Nordic countries	25 (3%)
Other European countries	92 (10%)
Asia	84 (9%)
Other continents	43 (5%)
Missing information	33 (4%)
Educational attainment	
Not completed high school	68 (8%)
Completed junior high school	162 (18%)
Completed senior high school	310 (35%)
Completed post-secondary education	263 (30%)
Missing information	78 (9%)
Medical comorbidities^1^, n (%)	170 (19%)

^1^ At least one clinical diagnosis of COPD, diabetes mellitus, CVD, thyroid disease, or kidney disease

Comparison between patients with complete and incomplete WHODAS-2.0 values revealed that patients with complete values had a lower mean age, but did not differ in sex, PANSS-8 sum score, living situation, or geographic birth area (Supplementary Table 1).

### Item score distribution

Parameters related to item score distributions are presented in Table 3. Generally, patients rated high on items that concerned difficulties participating in society and everyday life activities (S4, S12), and lower on items that concerned functional impairment in self-care (S8, S9). Unlike the summary score, two distributions of the two items were slightly skewed (S8, S9) and one item’s distribution showed a high kurtosis (S9). There was also evidence of floor effects regarding several items.

**Table 3:** 12-item WHODAS-2.0 item scores

	*Mean (SD*^*a*^*)*	*Median (range)*	*Floor*^*b*^	*Ceiling*^*c*^	*Skewness*	*Kurtosis*
*Item*						
S1 Standing long periods	1.80 (1.23)	1 (1–5)	63%	6%	1.36	0.60
S2 Household responsibilities	1.89 (1.10)	1 (1–5)	52%	3%	0.99	0.01
S3 Learning new tasks	1.90 (1.15)	1 (1–5)	53%	3%	1.04	−0.07
S4 Joining community activities	2.23 (1.31)	2 (1–5)	44%	6%	0.61	−0.97
S5 Emotionally affected	2.56 (1.20)	3 (1–5)	26%	3%	0.12	−1.19
S6 Concentrating	1.80 (1.10)	1 (1–5)	58%	2%	1.14	0.15
S7 Walking long distance	1.77 (1.25)	1 (1–5)	66%	6%	1.44	0.74
S8 Washing whole body	1.41 (0.92)	1 (1–5)	80%	2%	2.38	4.91
S9 Getting dressed	1.26 (0.71)	1 (1–5)	86%	1%	3.07	9.38
S10 Dealing with strangers	2.00 (1.17)	2 (1–5)	48%	3%	0.84	−0.48
S11 Maintaining friendships	1.94 (1.19)	1 (1–5)	53%	4%	1.01	−0.17
S12 Work/School activities	2.27 (1.44)	2 (1–5)	46%	12%	0.71	−0.92
WHODAS sum score	22.83 (9.38)	21 (12–56)	9%	0%	0.98	0.53

^a^ Standard deviation

^b^ % of the lowest possible score

^c^ % of highest possible score

### Confirmatory factor analysis

For the first model, one general disability factor was used as the latent factor for all 12 items. The model had a poor model fit (e.g., NC = 20.3, RMSEA = 0.15). Introducing the six IFC dimensions as first-order latent factors into model one yielded an improved, but still not acceptable model fit (e.g., NC = 9.4, RMSEA = 0.10).

**Fig. 1:**
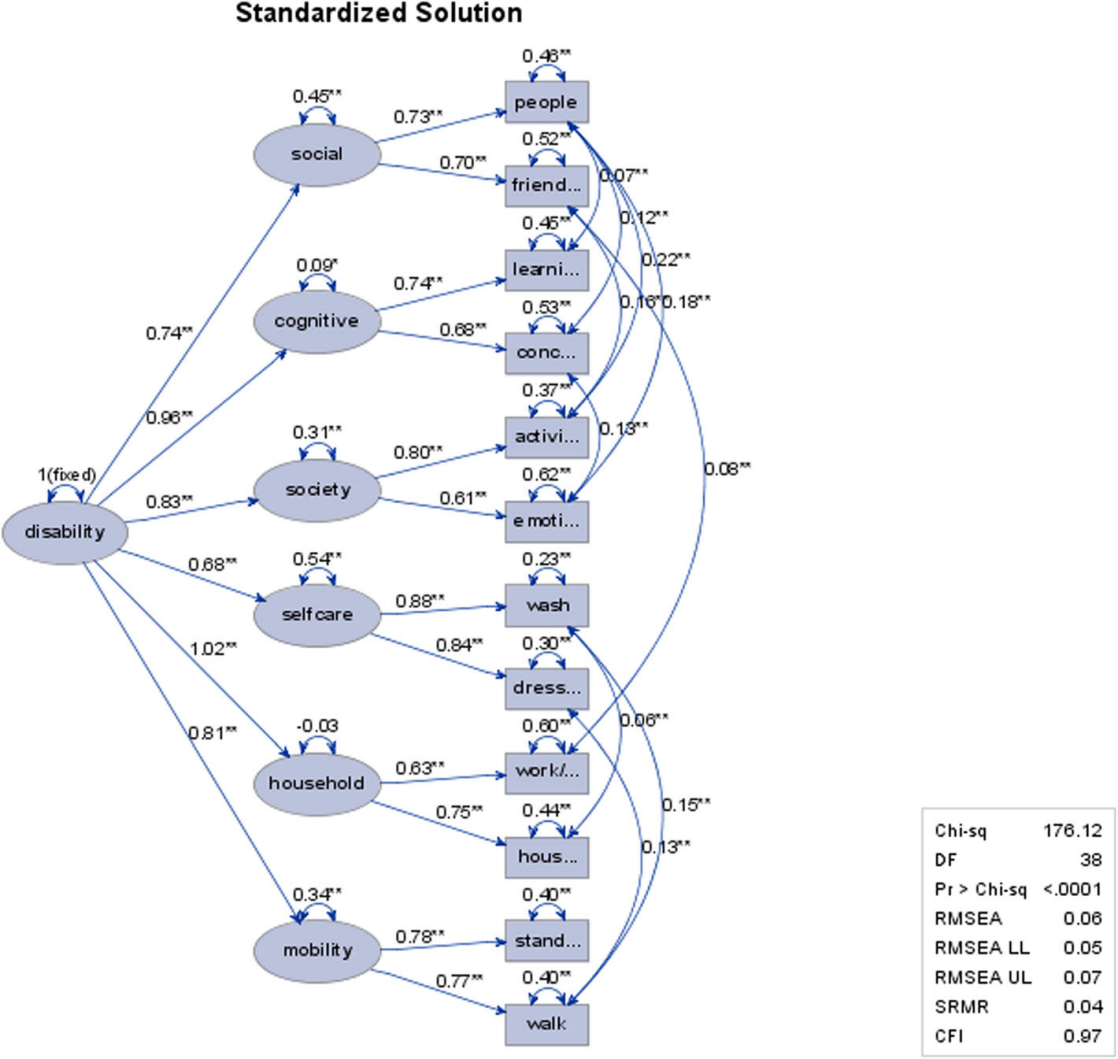
Adjusted two-level structure with one general disability factor and the six IFC dimensions as latent factors (*N* = 881). *p = < 0.05, **p = < 0.01

For the third model, we extended the second model by allowing for covariation between error terms until an acceptable model was obtained. The covariances made conceptual sense as they were identified within factors of a similar area of disability, i.e., physical disability (mobility, self-care, household), and socio-cognitive disability (cognitive, social, society). The following fit indices were yielded: NC = 4.8, CFI = 0.97, TLI = 0.945, SRMR = 0.05, and RMSEA = 0.07, indicating acceptable model fit (Fig. 1).

### Internal validity and reliability

All scale items showed good convergent and discriminant validity (Table 4).

**Table 4:** Item convergent and discriminant validity and internal reliability

Scale	No. of items / scale	Item convergent validity^**a**^	Item discriminant validity^**b**^	Cronbach’s alpha
Mobility	2	0.85, 0.87	0.32–0.51	0.77
Self-care	2	0.88, 0.95	0.28–0.50	0.84
Household	2	0.79, 0.89	0.33–0.54	0.63
Cognitive	2	0.83, 0.86	0.38–0.56	0.67
Social	2	0.88, 0.88	0.29–0.60	0.71
Society	2	0.86, 0.87	0.26–0.62	0.67
WHODAS-2.0	12			0.89

a Correlations with own scale.

b Correlations with other scales (range of correlations)

Internal reliability showed that the WHODAS-2.0 sum scale had near-to-excellent reliability, and the scale for self-care had good reliability. The scales for getting around and getting along showed acceptable reliability, and the scales for understanding and participation in society were close to acceptable. The scale for life activities showed questionable reliability. In Table 4, only Cronbach’s alpha scores are presented as the Spearman-Brown scores did not substantially differ.

### Objective measures associated with disability

To compare subgroups of age, the median of 53 years was used as the limit. The older group rated significantly higher in the total WHODAS-2.0 score than the younger group. Similarly, patients with a clinical diagnosis of COPD, diabetes mellitus, CVD, thyroid disease, or kidney disease had a significantly higher mean total score than patients without such diagnoses. Patients who utilized care assistance had higher mean total scores than patients who did not. Patients under no current antipsychotic medication had a lower mean total score than patients using such medication.

For the PANSS 8-item sum score, the median of 14 was used as the cut-off point. Patients with lower scores rated lower in the total WHODAS-2.0 sum score than patients with higher ones (Table 5).

**Table 5:** Mean values (SD) of WHODAS-2.0 sum score by categories of covariates associated with general disability

*Covariate*		*Disability categories*				
**Age**	Overall (*n* = 881)	Age < 53 (*n* = 429)	Age ≥ 53 (*n* = 452)	*p*-value^a^	Difference (95% CI)	Cohen’s d^b^
	22.8 (9.4)	21.8 (8.3)	23.8 (10.3)	0.002	1.9 (0.7, 3.2)	0.21
**Living situation**^c^	Overall (*n* = 857)	Independent (*n* = 477)	Assisted (*n* = 380)			
	22.7 (9.3)	20.2 (7.9)	25.9 (10.1)	< 0.0001	5.7 (4.5, 6.9)	0.60
**Medical** **comorbidity**^d^	Overall (n = 881)	No (*n* = 711)	Yes (*n* = 170)			
	22.8 (9.4)	22.5 (9.4)	24.2 (9.4)	0.03	1.7 (0.8, 3.3)	0.18
**Antipsychotic** **medication**	Overall (*n* = 843)	No (*n* = 61)	Yes (*n* = 782)			
	22.8 (9.3)	20.4 (7.6)	22.9 (9.4)	0.014	2.6 (0.2, 4.6)	0.28
**PANSS-8-items sum score**	Overall (*n* = 495)	Score ≤ 14 (*n* = 261)	Score > 14 (*n* = 234)			
	23.1 (9.2)	19.9 (8.0)	26.6 (9.0)	< 0.0001	6.8 (5.3, 8.3)	0.74

a) Two-sample t-test.

b) Difference in mean / pooled SD.

c) Independent = Living in own housing without assistance, Assisted = Living in own housing with assistance or in a group home.

d) At least one clinical diagnosis of COPD, diabetes mellitus, CVD, thyroid disease, or kidney disease

**Fig. 2:**
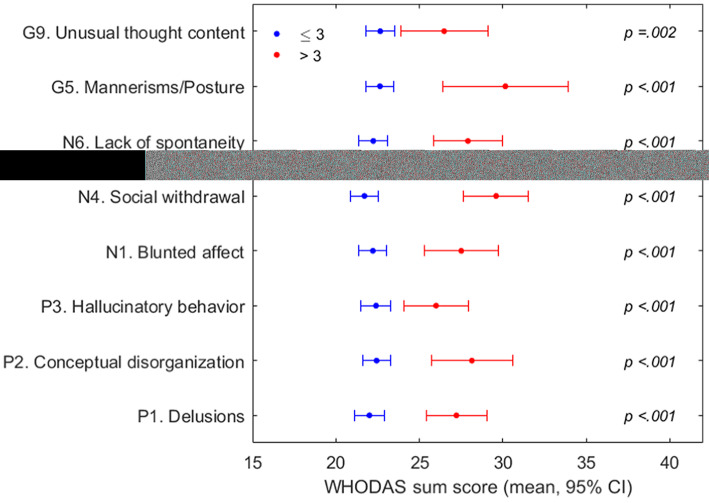
Differences in WHODAS-2.0 sum scores by categories of individual PANSS remission items defined as PANSS score ≤ 3 (mild to no psychotic symptoms) and PANSS score > 3 (moderate to extreme symptoms)

Using the remission score of 3 as the cut-off point, we investigated all 8 PANSS remission items and found that patients with higher rating on each PANSS item also had significantly higher WHODAS-2.0 sum scores (Fig. 2).

## Discussion

This study examined the clinical validity of the 12-item WHODAS-2.0 in a naturalistic sample of outpatients with psychotic disorders in order to inform clinical use of that assessment tool. We found that the sum scale showed near to excellent internal reliability and that construct validity was confirmed. Patients who were rated lower on PANSS-8 remission items generally also self-assessed at significantly lower WHODAS-2.0 sum scores than patients who were rated as having more symptoms.

We could confirm an adjusted two-level factor model with a single second-order disability factor and the six IFC dimensions as first-order factors. For a good model fit, covariances between error terms needed to be included for items representing physical disability (i.e., mobility, self-care, and household factors) and socio-cognitive disability (i.e., cognitive, social, and society factors). Further research should look at these separately as it is likely that the relative importance of these two aspects could differ for different clinical populations, e.g., patients with primarily mental or physical diseases.

All scale items showed adequate convergent and discriminant validity. However, floor effects were observed for many of the items. This might be due to selection effects since not all enlisted outpatients participate at the annual check-ups and those who participate might be more functional than non-participants. However, the floor effects were similar to a Swedish research study evaluating the WHODAS-2.0 12-item version in patients with anxiety disorders [14]. If floor effects are present, it is likely that values are missing in the upper end of the scale. Consequently, it might be difficult to discriminate between patients, reducing the score’s reliability [30]. Floor effects and poor internal reliability were evident for three subscales, but not for the sum score, suggesting that the sum score is more robust than individual item scores.

In terms of internal reliability, the scale for household was questionable. Item S12, asking about day-to-day work/school, was the item with the most missing values during data collection. This is probably related to our clinical sample including several patients not working or studying (e.g., on disability pension or retired). However, most patients in the sample above the Swedish retirement age of 65 had values for S12, and it is difficult to know how patients and clinicians interpreted this item. According to the WHODAS-2.0 manual, this global question is intended to elicit respondents’ appraisal of difficulties encountered in day-to-day work or school activities, such as being on time, planning, and organizing. Statistically, this item did not differ substantially from other items, except that the mean score was somewhat higher than most other items with relatively low floor effects. Because this item does not refer to a concrete activity for patients that neither work nor study, it might be interpreted in different ways in particular, when case managers are not updated on the WHODAS-2.0 manual. Future research should use qualitative methods to explore the interpretation of this item, from clinicians’ and patients’ perspectives, particularly in relation to patients who do not work or study.

Patients not on antipsychotic medication generally rated lower on the WHODAS-2.0. One potential explanation for this can be that these patients might experience more symptoms and thus overestimate their function. However, we did not see such tendencies in this study as patients who were rated high on the remission items (more severe symptoms) also rated themselves high on WHODAS-2.0 (more functional impairment). It is likely that patients not on antipsychotic medication do not have debilitating symptoms that fully warrant medication or that they do not experience the well-known side effects and subsequent function impairment associated with treatment [5]. However, we must be cautious when interpretating this finding and consider that we lacked significant information about the participating patients which might influence this outcome. We lacked information on what specific psychotic disorder the patients were diagnosed with, their psychiatric comorbidities, and what other type of medication they were using. Thus, there might be variations in psychiatric comorbidities which influenced the treatment rationales beyond psychotic symptomatology as well as polypharmaceutical effects that we were unable to account for.

Several clinical implications can be highlighted from this study. The confirmation of an adjusted factor structure that used the six IFC dimensions as subscales indicates that it is possible to also use these domains for the WHODAS-2.0 12-item instrument. This applies particularly to the factors of mobility, self-care, and social, which showed acceptable internal reliability. This result is important as there is a need for schizophrenia research to shift its focus – from an excessive reliance on global measures of psychopathology and disability to the creation of profiles of specific psychosocial disabilities [5]. Systematic reviews show that the two common global symptom rating scales, PANSS and BPRS, have limited value as outcome measures for functioning in practical settings. Instead, to get a better understanding of patients’ everyday functioning and needs, we should consider several domains including cognition and personal and social functioning [9, 31].

An important issue considering the clinical implications is that the WHODAS-2.0 does not currently assess environmental factors. According to the IFC [32], environmental factors are defined as the contextual characteristics that make up the attitudinal, physical and social environment in which individuals live. Important factors can be assistive technologies to compensate for disability such as hearing aids and glasses or building design factors influencing a person’s mobility. In the WHODAS-2.0 manual [19], it is suggested that the clinician can add probing questions to ask about environmental factors where any difficulty is reported in the current WHODAS-2.0. While this addition might add to the time of the administration, it would be particularly valuable for patients with particular needs. A related clinical aspect is the consideration of the high prevalence of chronic diseases among patients with psychotic disorders. Thus, regarding the domain ‘self-care’ it would be useful to add a question on the ability of self-management of chronic diseases, such as diabetes. Another aspect related to the clinical usage is how frequently to assess patients’ function with the WHODAS-2.0. At our clinics' , it is administered as part of the annual check-ups but considering that the instrument intends to assess impairment over the last 30 days, a more frequent assessment might be preferable to e.g. monitor the patients’ response to treatment or detect deteriorations.

### Strength and limitations

Several strengths and limitations have been identified in this study. An important strength is the inclusion of a clinically representative sample, demonstrated by patients’ wide age range, different living situations, and medical comorbidities. The inclusion of older patients is important as older adults are often underrepresented in clinical research [33]. However, it must be noted that the two outpatient clinics used for data collection might not be fully representative of all the outpatient clinics, as we know that these clinics have a higher proportion of patients born in Sweden compared to other outpatient clinics at the hospital where this study was conducted. Patients with migrant backgrounds might face additional challenges to accessing mental health services due to language barriers and a greater variation in healthcare literacy. Recent Swedish research showed higher rates of involuntary admission in neighborhoods with higher migrant density, suggesting that there may be sociocultural barriers affecting timely access to mental health care [34]. Further, these two clinics are also situated in areas with a relatively high socioeconomic status compared to some of the other outpatient clinics.

The WHODAS-2.0 is a self-assessed measure, but it is often modified in dialogue between the patient and clinicians [10]. A limitation is that we do not know which observations were modified and to what extent. Another limitation is that we lacked information about the specific diagnoses of the patients, their psychiatric comorbidities, and what other types of medications the patients were treated with besides antipsychotics, such as mood stabilizers, anxiolytic medications, or antidepressants. Some of these medications might have similar effects as some antipsychotics, thus influencing symptom severity and function. It is also a limitation that we did not have any performance-based measures, such as the UCSD Performance-based Skills Assessment, as these measures might provide a less biased assessment of patients’ functioning [35].

## Conclusions

This study shows the clinical significance of the WHODAS-2.0 (12-item) in a naturalistic sample of outpatients with psychotic disorders by demonstrating strong relationships with patients’ age, clinically diagnosed comorbidities, living situation, antipsychotic medication status, and symptom severity. Few previous studies have evaluated the 12-item WHODAS-2.0 construct validity in subgroups of clinically diagnosed disease-specific parameters [2]. This is valuable since self-assessed methods are prone to subjectivity and reporting bias, and to correlate only self-assessed measures might lead to improper evaluation of construct validity. Thus, this study confirms the instrument’s clinical validity, such that patients’ clinical characteristics were visibly differentiated in the 12-item WHODAS-2.0 sum scores. However, we should still consider this with caution, first because we do not know to what extent the WHODAS-2.0 scores were modified by clinicians, and second because previous research suggests that many patients with schizophrenia tend to misjudge their everyday functioning [35]. Research focusing on the accuracy of self-estimation conclude that individuals with schizophrenia, on average, overestimate their function [35]. Still, the same study also showed that the accuracy of self-estimated function was influenced by patients’ psychiatric comorbidity and symptomatology. They found that patients with poorer neuropsychological performance and functional capacity tended to overestimate their everyday functioning. In contrast, they found that higher levels of depression were associated with a more accurate self-assessment.

The wider use of the 12-item WHODAS-2.0 instrument might help capture the subjective experience of the patients and thus narrow the potential gaps between clinicians’ and patients’ perspectives. By showing that clinician-rated symptom remission follows the WHODAS-2.0 sum score, the clinical validity of this self-assessed instrument is demonstrated.

## Supplementary Information

**Additional file 1.**

## Data Availability

The data that support the findings of this study are available by reasonable request from the corresponding author (CH). The data are not publicly available due to their containing information that could compromise patients’ privacy.

## Abbreviations

CFI: Comparative fit index
DF: Degrees of freedom
ICF: International Classification of Functioning, Disability, and Health
SD: Standard deviation
WHO: World Health Organization
WHODAS: World Health Organization Disability Assessment Schedule

## References

[1] Üstün TB, Chatterji S, Kostanjsek N, Rehm J, Kennedy C, Epping-Jordan J (2010). Developing the World Health Organization disability assessment schedule 2.0. Bull World Health Organ.

[2] Federici S, Bracalenti M, Meloni F, Luciano JV (2017). World Health Organization disability assessment schedule 2.0: an international systematic review. Disabil Rehabil.

[3] Strassnig M, Kotov R, Cornaccio D, Fochtmann L, Harvey PD, Bromet EJ (2017). Twenty-year progression of body mass index in a county-wide cohort of people with schizophrenia and bipolar disorder identified at their first episode of psychosis. Bipolar Disord.

[4] Annamalai A, Kosir U, Tek C (2017). Prevalence of obesity and diabetes in patients with schizophrenia. World J Diabetes.

[5] Świtaj P, Anczewska M, Chrostek A, Sabariego C, Cieza A, Bickenbach J (2012). Disability and schizophrenia: a systematic review of experienced psychosocial difficulties. BMC Psychiatry.

[6] Strassnig M, Signorile J, Gonzalez C, Harvey PD (2014). Physical performance and disability in schizophrenia. Schizophr Res Cogn.

[7] Nelson EC, Eftimovska E, Lind C, Hager A, Wasson JH, Lindblad S. Patient reported outcome measures in practice. BMJ [Internet]. 2015 10 [cited 2020 Sep 29];350. Available from: https://www.bmj.com/content/350/bmj.g7818 10.1136/bmj.g7818 25670183

[8] Black N. Patient reported outcome measures could help transform healthcare. BMJ [Internet]. 2013 28 [cited 2020 Sep 29];346. Available from: https://www.bmj.com/content/346/bmj.f167 10.1136/bmj.f167 23358487

[9] Mortimer AM (2007). Symptom rating scales and outcome in schizophrenia. Br J Psychiatry.

[10] Gspandl S, Peirson RP, Nahhas RW, Skale TG, Lehrer DS (2018). Comparing global assessment of functioning (GAF) and World Health Organization disability assessment schedule (WHODAS) 2.0 in schizophrenia. Psychiatry Res.

[11] Andrews G, Kemp A, Sunderland M, Von Korff M, Ustun TB. Normative Data for the 12 Item WHO Disability Assessment Schedule 2.0. Ross JS, editor. PLoS ONE. 2009 Dec 17;4(12):e8343. 10.1371/journal.pone.0008343 PMC2791224 20020047

[12] Weeks M, Garber BG, Zamorski MA. Disability and Mental Disorders in the Canadian Armed Forces. Can J Psychiatry. 2016 Apr;61(1_suppl):56S–63S. 10.1177/0706743716628853 PMC4800473 27270743

[13] McEvoy PM, Mahoney AEJ (2011). Achieving certainty about the structure of intolerance of uncertainty in a treatment-seeking sample with anxiety and depression. J Anxiety Disord.

[14] Axelsson E, Lindsäter E, Ljótsson B, Andersson E, Hedman-Lagerlöf E. The 12-item Self-Report World Health Organization Disability Assessment Schedule (WHODAS) 2.0 Administered Via the Internet to Individuals With Anxiety and Stress Disorders: A Psychometric Investigation Based on Data From Two Clinical Trials. JMIR Ment Health. 2017 Dec 8;4(4):e58. 10.2196/mental.7497 PMC5741825 29222080

[15] Swedish National Board of Health and Welfare. Mätning av hälsa och funktionshinder. Manual till WHO:s formulär för bedömning av funktionshinder WHO Disability Assessment Schedule [Eng: Measuring health and disability. Manual for WHO Disability Assessment Schedule] [Internet]. Stockholm, Sweden; 2015 [cited 2021 Jan 7] p. 134. Available from: https://www.socialstyrelsen.se/globalassets/sharepoint-dokument/artikelkatalog/ovrigt/2015-5-1.pdf

[16] Fagiolini A, Rocca P, De Giorgi S, Spina E, Amodeo G, Amore M (2017). Clinical trial methodology to assess the efficacy/effectiveness of long-acting antipsychotics: randomized controlled trials vs naturalistic studies. Psychiatry Res.

[17] Brink M, Green A, Bojesen AB, Lamberti JS, Conwell Y, Andersen K (2019). Excess medical comorbidity and mortality across the lifespan in schizophrenia. Schizophr Res.

[18] Gremyr A, Gäre BA, Greenhalgh T, Malm U, Thor J, Andersson A-C (2020). Using complexity assessment to inform the development and deployment of a digital dashboard for schizophrenia care: case study. J Med Internet Res.

[19] Üstün TB, Editor. Measuring health and disability: manual for WHO disability assessment schedule WHODAS 2.0. Geneva: World Health Organization; 2010. 90 p.

[20] Andreasen NC, Carpenter WT, Kane JM, Lasser RA, Marder SR, Weinberger DR (2005). Remission in schizophrenia: proposed criteria and rationale for consensus. Am J Psychiatry.

[21] Liechti S, Capodilupo G, Opler DJ, Opler M, Yang LH (2017). A developmental history of the positive and negative syndrome scale (PANSS). Innov Clin Neurosci.

[22] Lambert M, Karow A, Leucht S, Schimmelmann BG, Naber D (2010). Remission in schizophrenia: validity, frequency, predictors, and patients’ perspective 5 years later. Dialogues Clin Neurosci.

[23] Os J, Burns T, Cavallaro R, Leucht S, Peuskens J, Helldin L (2006). Standardized remission criteria in schizophrenia. Acta Psychiatr Scand.

[24] Hu L, Bentler PM (1999). Cutoff criteria for fit indexes in covariance structure analysis: conventional criteria versus new alternatives. Struct Equ Model Multidiscip J.

[25] West SG, Finch JF, Curran PJ. Structural equation models with nonnormal variables: Problems and remedies. In: Structural equation modeling: Concepts, issues, and applications. Thousand Oaks, CA, US: Sage Publications, Inc; 1995. p. 56–75.

[26] Schumacker RE, Lomax RG. A beginner’s guide to structural equation modeling. 3rd ed. New York: Routledge; 2010. 510 p.

[27] Portney LG, Watkins MP. Foundations of clinical research: applications to practice. 3rd ed. Upper Saddle River, N.J: Pearson/Prentice Hall; 2009. 892 p.

[28] Eisinga R, te Grotenhuis M, Pelzer B (2013). The reliability of a two-item scale: Pearson, Cronbach, or spearman-Brown?. Int J Public Health.

[29] Lance CE, Butts MM, Michels LC (2006). The sources of four commonly reported cutoff criteria: what did they really say?. Organ Res Methods.

[30] Terwee CB, Bot SDM, de Boer MR, van der Windt DAWM, Knol DL, Dekker J (2007). Quality criteria were proposed for measurement properties of health status questionnaires. J Clin Epidemiol.

[31] Harvey PD, Strassnig MT, Silberstein J (2019). Prediction of disability in schizophrenia: symptoms, cognition, and self-assessment. J Exp Psychopathol.

[32] International Classification of Functioning, Disability and Health (ICF) [Internet]. [cited 2021 Jan 16]. Available from: https://www.who.int/standards/classifications/international-classification-of-functioning-disability-and-health

[33] Herrera AP, Snipes SA, King DW, Torres-Vigil I, Goldberg DS, Weinberg AD (2010). Disparate inclusion of older adults in clinical trials: priorities and opportunities for policy and practice change. Am J Public Health.

[34] Terhune J, Dykxhoorn J, Mackay E, Hollander A-C, Kirkbride JB, Dalman C (2020). Migrant status and risk of compulsory admission at first diagnosis of psychotic disorder: a population-based cohort study in Sweden. Psychol Med.

[35] Sabbag S, Twamley EW, Vella L, Heaton RK, Patterson TL, Harvey PD (2012). Predictors of the accuracy of self assessment of everyday functioning in people with schizophrenia. Schizophr Res.

