# Supplementary material for: Clinical validity of the 12-item WHODAS-2.0 in a naturalistic sample of outpatients with psychotic disorders
Source: BMC Psychiatry. 2021 Mar 10;21:147. doi: 10.1186/s12888-021-03101-9 (PMC7945302; doi:10.1186/s12888-021-03101-9)
Supplement: Supplementary file 1 — Additional file 1. [file 12888_2021_3101_MOESM1_ESM.docx]

# Manuscript title

Clinical validity of the 12-item WHODAS-2.0 in a Naturalistic Sample of Outpatients with Psychotic Disorders

Authors

Christopher Holmberg^1,2^, Andreas Gremyr^1,3^, Jarl Torgerson^2^, Kirsten Mehlig^4^

1. Institute of Health and Care Sciences, University of Gothenburg, Sweden
2. Department of Psychotic Disorders, Sahlgrenska University Hospital, Gothenburg, Sweden
3. Jönköping Academy for Improvement of Health and Welfare, Jönköping University, Jönköping, Sweden
4. School of Public Health and Community Medicine, Institute of Medicine, University of Gothenburg, Sweden

Author emails

- [christopher.holmberg@gu.se](mailto:christopher.holmberg@gu.se)
- [andreas.gremyr@vgregion.se](mailto:Axel.wolf@gu.se)
- jarl.torgerson@vgregion.se
- kirsten.mehlig@gu.se

Corresponding author

Christopher Holmberg, RN, PhD, Assistant Professor

Email: [christopher.holmberg@gu.se](mailto:christopher.holmberg@gu.se)

Address: Institute of Health and Care Sciences, University of Gothenburg. Arvid Wallgrens Backe, Box 457, 405 30, Göteborg, Sweden. Tel: +46 (0) 766-18 18 52.

&

Department of Psychotic Disorders, Sahlgrenska University Hospital, Gothenburg, Sweden.

# Supplementary file 1

Figure S1. Sampling flowchart and sample comparison statistics.

All registered WHODAS-2.0 observations at the two outpatient clinics between 2016–2019 (n=1347)

Observations with missing WHODAS-2.0 values (n=225)

Observations with complete WHODAS-2.0 values (n=1122)

Including the first registered WHODAS-2.0 values per patient, thus excluding all other observations (n=241)

The first registered WHODAS-2.0 values from a unique patient (n=881)

| Table S1. Dropout analysis. | | | | |  |
| --- | --- | --- | --- | --- | --- |
| **Characteristics** |  | **Sample (n=881)^1^** | | | **Sample (n=183)^2^** |
|  |  |  | | |  |
| Age (years), mean (SD), range |  | 52 (13.9), 20–92 | | | 58 (12.4), 21–93^*^ |
| Female, n (%) |  | 413 (47%) | | | 91 (50%) |
|  |  |  | | |  |
| PANSS-8-items sum score |  |  | | |  |
| Mean (SD), range |  | 15.52 (6.9), 8–37 | | | 15.26 (9.4), 8–46 |
|  |  |  | | |  |
| Living situation, n (%) |  |  | | |  |
| Independently in regular housing |  | 477 (54%) | | | 58 (32%) |
| Missing values |  | 24 (3%) | | | 68 (37%) |
|  |  |  | | |  |
| Geographic birth area, n (%) |  |  | | |  |
| Sweden |  | 604 (69%) | | | 75 (41%) |
| Missing values |  | 33 (4%) | | | 68 (37%) |
|  | | |  |  |  |
| ^1^ Unique patients with complete WHODAS-2.0  ^2^ Unique patients with incomplete WHODAS-2.0 not included in main analyses  ^*^ Significant difference at 0.05-level (t-test) | | | | | |
|  | | |  |  |  |
